# Supplementary material for: Atomic electrostatic maps of 1D channels in 2D semiconductors using 4D scanning transmission electron microscopy
Source: Nat Commun. 2019 Mar 8;10:1127. doi: 10.1038/s41467-019-08904-9 (PMC6408534; doi:10.1038/s41467-019-08904-9)
Supplement: Supplementary file 1 — Supplementary Information [file 41467_2019_8904_MOESM1_ESM.pdf]

Atomic Electrostatic Maps of 1D Channels in 2D Semiconductors using 4D Scanning Transmission Electron Microscopy

Shiang Fang[1†], Yi Wen[2†], Christopher S. Allen[2,3], Colin Ophus[4], Grace G. D. Han[5], Angus I. Kirkland[2,3], Efthimios Kaxiras[1,6], Jamie H. Warner[2*]*

[1] *Department of Physics, Harvard University, Cambridge, Massachusetts 02138, USA*

[2]*Department of Materials, University of Oxford, 16 Parks Road, Oxford OX1 3PH, United Kingdom*

[3]*Electron Physical Sciences Imaging Center, Diamond Light Source Ltd., Didcot, Oxfordshire, OX11 0DE, United Kingdom*

[4]*National Center for Electron Microscopy, Molecular Foundry, Lawrence Berkeley National Laboratory, 1 Cyclotron Road, Berkeley, CA, USA.*

[5]*Department of Chemistry, Brandeis University, Waltham, Massachusetts, USA*

[6]*John A. Paulson School of Engineering and Applied Sciences, Harvard University, Cambridge, Massachusetts 02138, USA*

[†]*These authors contributed equally, * These authors jointly supervised this work*

**Email: Jamie.warner@materials.ox.ac.uk; kaxiras@physics.harvard.edu;*

Supplementary Information

Supplementary Figures

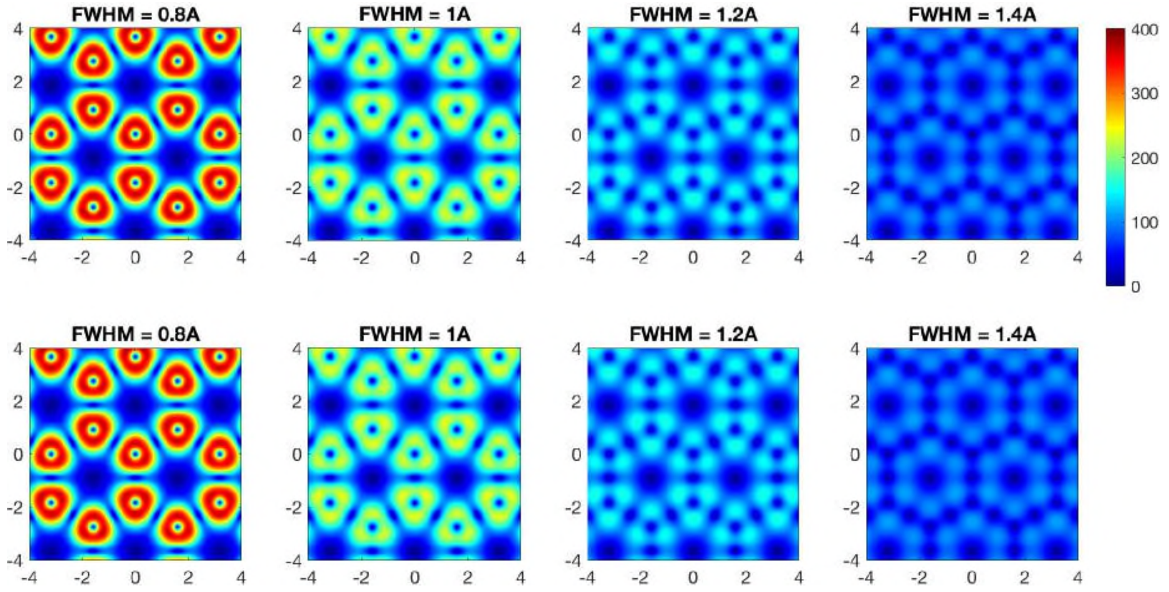

Supplementary figure 1. MoS_2 pristine monolayer crystal: integrated electric field $\tilde{E}^{\sigma}(\vec{r})$ calculated from pseudo-potential DFT (upper figures) and all-electron DFT (lower figures) with varying FWHM (from 0.8Å to 1.5Å) for gaussian smearing. The unit is in volt.

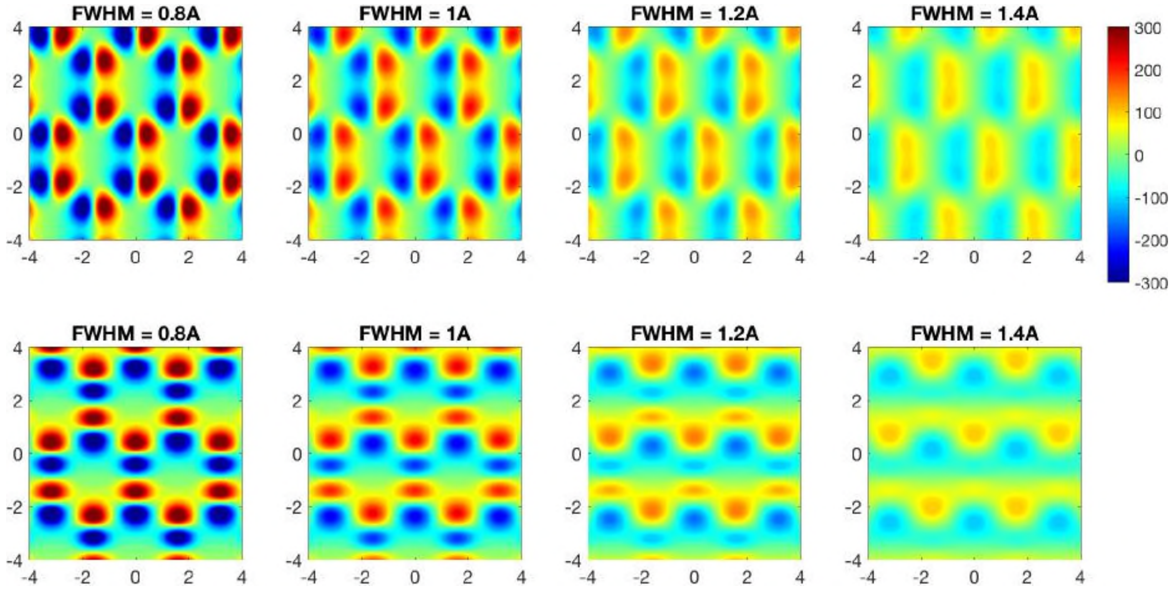

Supplementary figure 2: MoS_2 pristine monolayer crystal: integrated electric field $\tilde{E}_x^{\sigma}(\vec{r})$ (upper figures) and $\tilde{E}_y^{\sigma}(\vec{r})$ (lower figures) with varying FWHM (from 0.8Å to 1.4Å) for gaussian smearing. The unit is in volt.

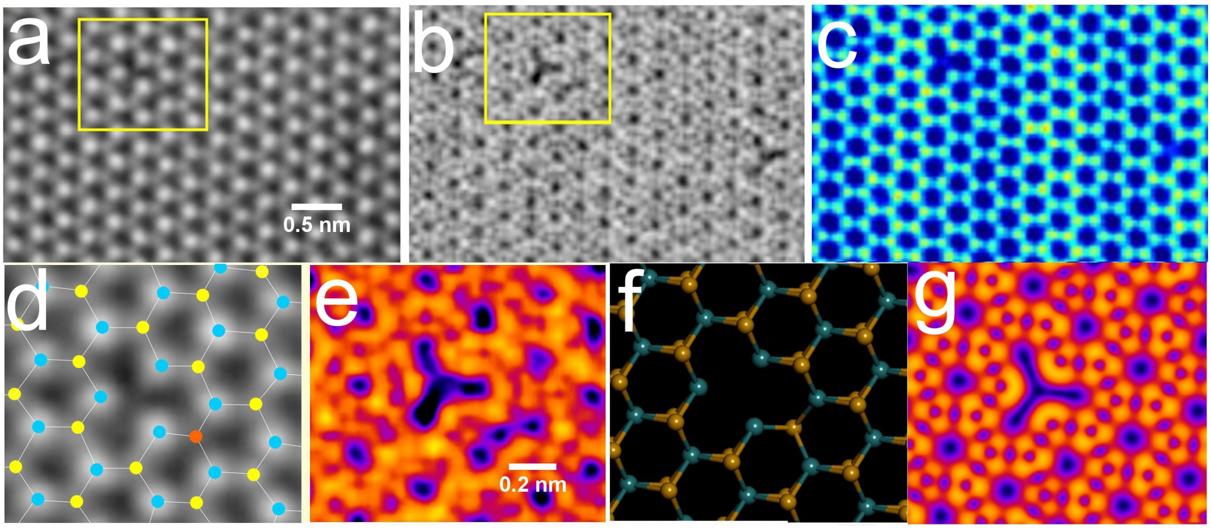

Supplementary figure 3. 4D STEM of S point vacancies in MoS_2. (a) LAADF image reconstructed from the 4D STEM data. (b) Experimental $|E_\perp|$ around MoS_2 reconstructed from the 4D STEM

data, using the modulus of I_{com}, normalized and scaled to match the DFT data range. (c) Ptychographic phase of the same area reconstructed from the 4D STEM data. (d) Magnified view from the yellow box in (a). Orange spot indicates 1S vacancy position. (e) Magnified view of the yellow box in (b), with a colour LUT (Fire). (f) Atomic model of 2S point vacancy. (g) DFT calculated $|E_\perp|$ map around a 2S defect in MoS_2.

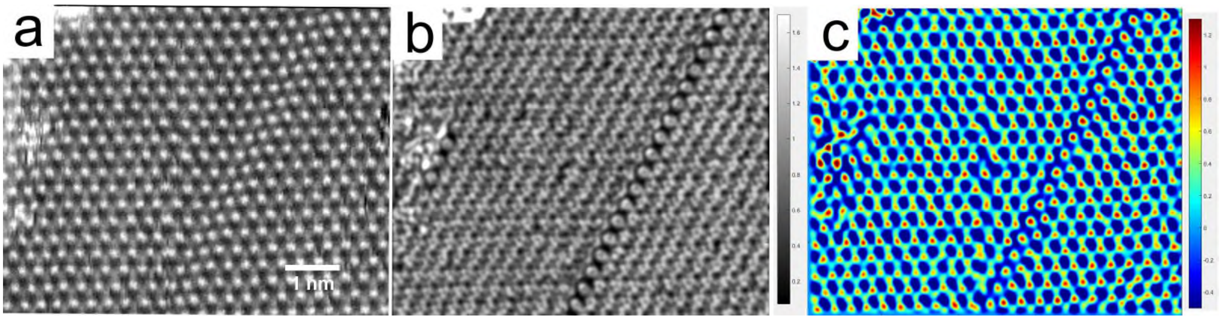

Supplementary figure 4. 4D STEM data of 2S line vacancy in WS_2. (a) (a) LAADF image reconstructed from the 4D STEM data. (b) Experimental $|E_\perp|$ around WS_2 reconstructed from the 4D STEM data, using the modulus of I_{com}, normalized and scaled to match the DFT data range. (c) Total charge image reconstructed from the 4D STEM data. Colour LUT used.

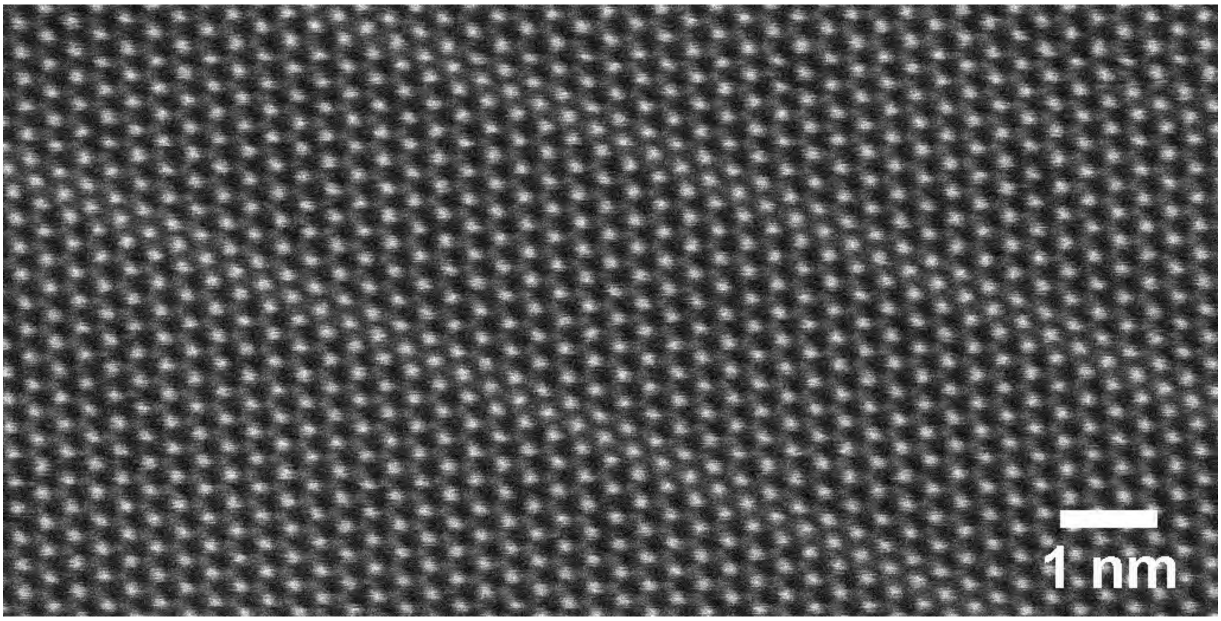

Supplementary figure 5. ADF-STEM image of two isolated ultralong S line vacancines in WS_2.

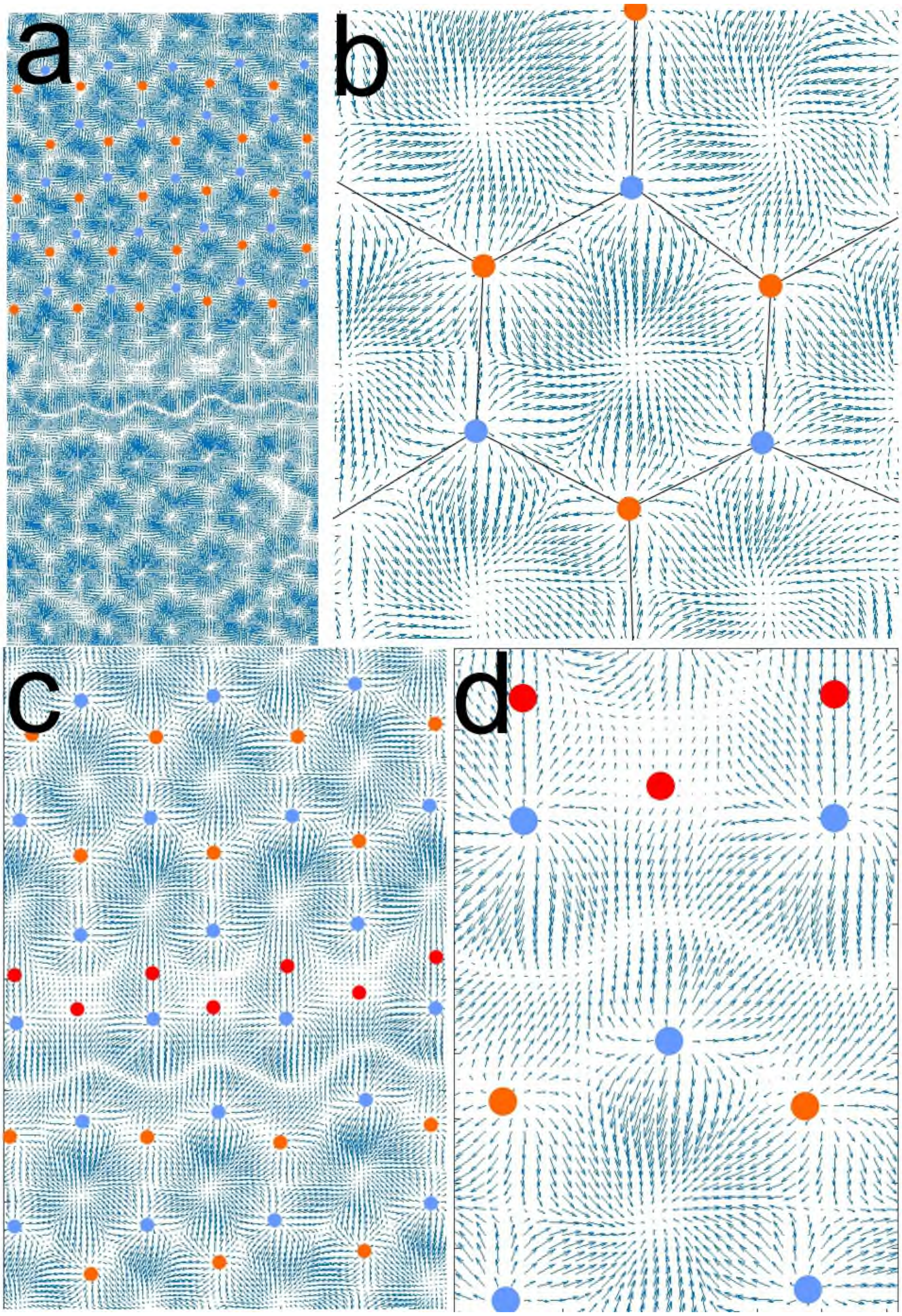

a
b
c
d

Supplementary figure 6. (a) Vector map of E⊥ around a line vacancy in MoS_2. (b) Higher magnification map of a pristine area. (c) Higher magnification image of the line defect area. (d) Higher magnification image of the 1D channel.

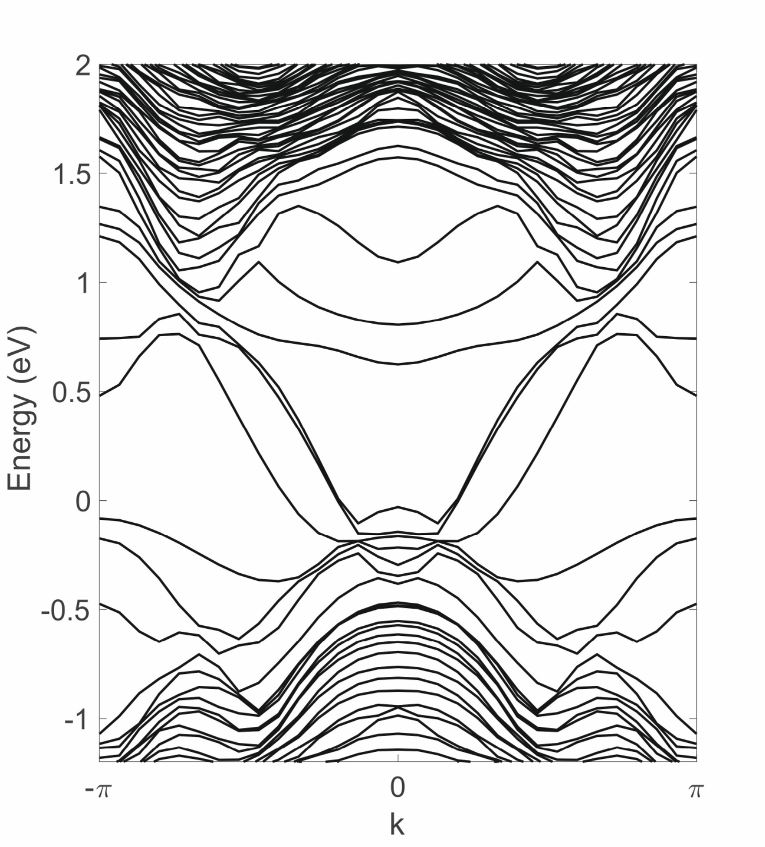

Supplementary figure 7. DFT calculated band structure of in-gap states for a 2SVL line vacancy in WS_2.

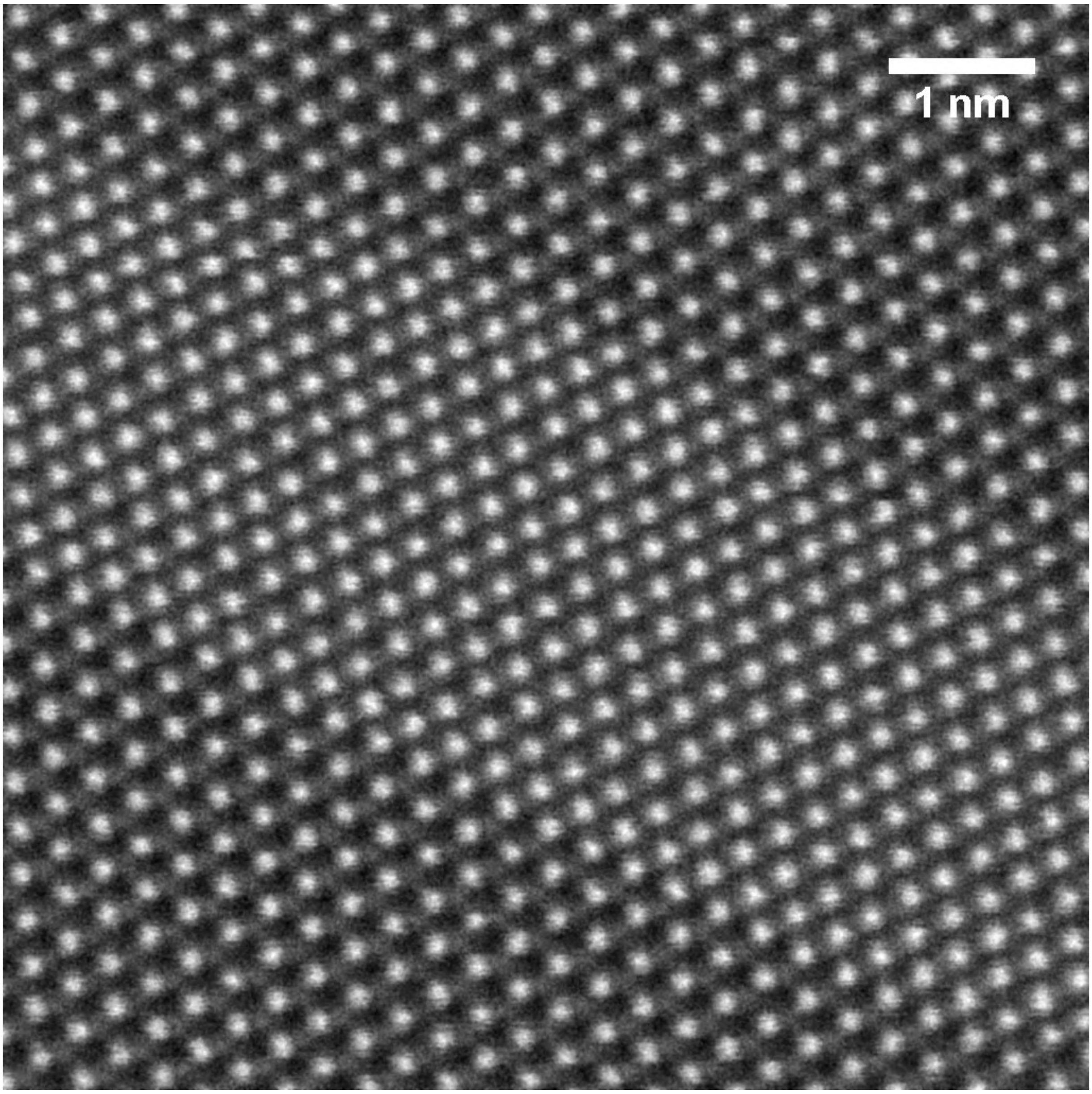

Supplementary figure 8. ADF-STEM image recorded with a long scan dwell time (32 μs per pixel) of a complex line vacancy regions in WS_2 at 800°C.

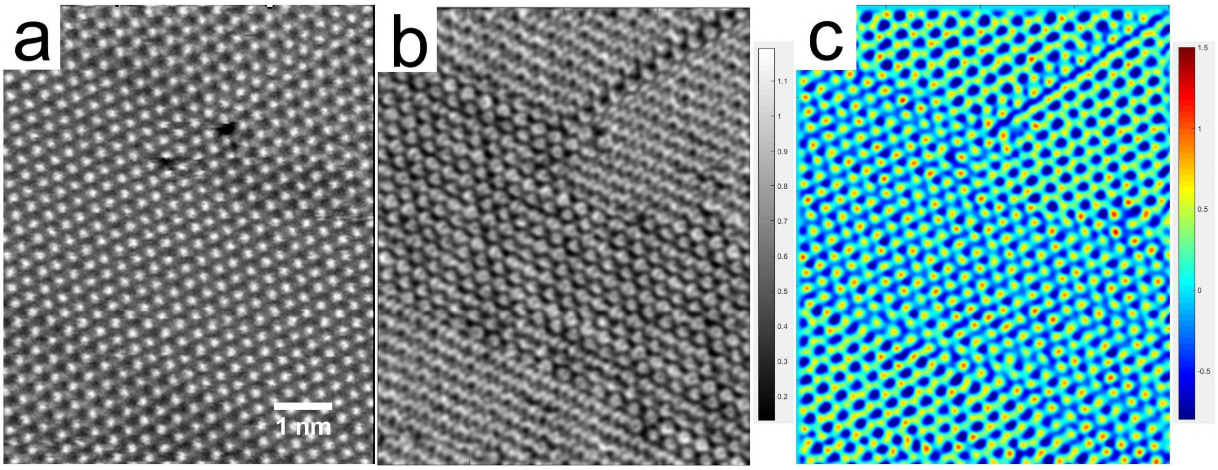

Supplementary figure 9. 4D STEM data of dense vacancy area in WS_2. (a) LAADF image reconstructed from the 4D STEM data. (b) Experimental |E⊥| around WS_2 reconstructed from the 4D STEM data, using the modulus of I_{com}, normalized and scaled to match the DFT data range. (c) Total charge image reconstructed from the 4D STEM data. Colour LUT used.

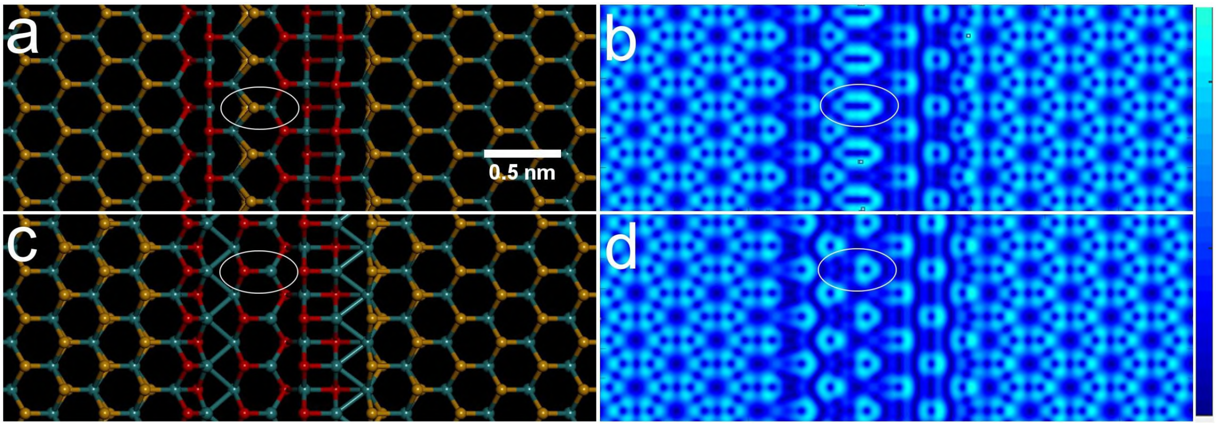

Supplementary figure 10. Comparison of DFT calculated |E⊥| in MoS_2 for two slightly different defect structures in MoS_2. (a) and (c) are atomic models and (b),(d) the respective DFT calculations. White oval indicates region where number of S atoms changes. Red atoms indicate 1S sites, due to S vacancies. Orange atoms are S in 2S sites and blue atoms are Mo.

Supplementary Note 1

The following matlab code shows the implementation of computing the FFT component with smearing.

```
%MATLAB code for DFT using 1.2 sigma smearing.

%% define constants

ep0=8.854187817E-12; % F/m
echarge=1.6E-19; % C
AAunit=1E-10;
EF_convert=echarge*(1/(AAunit^2))/ep0/(1/AAunit);
%% compute the FFT component with smearing
EF_smear_fft=zeros(num_hex,2);
valence_CHARGE_smear_fft=zeros(num_hex,1);
total_CHARGE_smear_fft=zeros(num_hex,1);
beam_sigma_constant=1/2/sqrt(2*log(2));
beam_sigma=(1.2)*beam_sigma_constant;

for indh=1:num_hex
    indh
    bvec_now=hex_list(indh,:);
    total_rho_fft=total_charge_fft(indh);
    nbvec_now=bvec_now/sqrt(dot(bvec_now,bvec_now));
    gauss_fac=exp(-dot(bvec_now,bvec_now)*beam_sigma*beam_sigma/2);

    EF_smear_fft(indh,1:2)=-
i*total_rho_fft*nbvec_now/sqrt(dot(bvec_now,bvec_now))*gauss_fac;
    valence_CHARGE_smear_fft(indh)=-valence_charge_fft(indh)*gauss_fac;
    total_CHARGE_smear_fft(indh)=total_charge_fft(indh)*gauss_fac;

end
%% Fourier transform back to real space for the charge and electric field
(smeared)
num1=1000;
num2=100;
dis_x=linspace(0,42,num1)+all_atom_pos_shift(1);
dis_y=linspace(-7,7,num2)+all_atom_pos_shift(2);
[disp_xx,disp_yy]=meshgrid(dis_x,dis_y);
smear_EF=zeros(num2,num1,2);
smear_valence_charge=zeros(num2,num1);
smear_total_charge=zeros(num2,num1);

for indh=1:num_hex
    indh
    bvec_now=hex_list(indh,:);

    tmpexp=exp(i*(disp_xx*bvec_now(1)+disp_yy*bvec_now(2)));
```

```
    smear_EF(:,:,1)=smear_EF(:,:,1)+EF_smear_fft(indh,1)*tmpexp;
    smear_EF(:,:,2)=smear_EF(:,:,2)+EF_smear_fft(indh,2)*tmpexp;

smear_valence_charge(:,:)=smear_valence_charge(:,:)+valence_CHARGE_smear_fft(
indh)*tmpexp;

smear_total_charge(:,:)=smear_total_charge(:,:)+total_CHARGE_smear_fft(indh)*
tmpexp;
end

smear_valence_charge=smear_valence_charge-valence_charge_fft(num_hex+1);

smear_EF=real(smear_EF)*EF_convert;
smear_valence_charge=real(smear_valence_charge);
smear_total_charge=real(smear_total_charge);
```
